# Robotic Total Knee Replacement: Single-Centre, Prospective, Non-Randomised Comparative Study Comparing Restricted Kinematic Alignment Combined with a Load Sensor Versus Functional Alignment

**DOI:** 10.3390/jcm15041396

**Published:** 2026-02-10

**Authors:** César Tourtoulou, Julien Bardou-Jacquet, François Blaquière, Nicolas Pommier, Pierre Laumonerie, Jérôme Murgier, Yohan Legallois

**Affiliations:** 1 Service Chirurgie Orthopédique et Traumatologique, CHU Bordeaux Pellegrin, Place Amélie Raba-Léon, 33000 Bordeaux, France; 2 Clinique Tivoli-Ducos, Institut de Chirurgie Robotique Euratlantique, 220, Rue Mandron, 33000 Bordeaux, France; 3 Service de Chirurgie Orthopédique, Ramsay Santé, Clinique Aguiléra 21 Rue de L’Estagnas, 64200 Biarritz, France

**Keywords:** robotic surgery, total knee arthroplasty, load sensor, kinematic alignment

## Abstract

**Background**: Total knee arthroplasty (TKA) is an effective procedure for symptomatic end-stage knee arthritis with good clinical and survivorship outcomes. However, up to 20% of patients report dissatisfaction following TKA. Recent studies have suggested that this may be at least partially due to suboptimal limb alignment or ligament imbalance. This study compared clinical outcomes at 1 year post-operatively (i.e., the 2011 Knee Society Score [KSS] and Forgotten Joint Score [FJS]) between two robotic-assisted personalised TKA techniques: functional alignment (FA) and an original technique combining restricted kinematic alignment (rKA) with a load sensor to achieve reliable ligament balancing (via bone re-cutting with a robotic arm). **Methods**: This single-centre, prospective, comparative study was performed at a robotic-assisted arthroplasty centre. The study population consisted of an FA group (43 patients) and rKA/sensor group (47 patients). Clinical outcomes were measured at 1 month post-operatively (visual analogue scale [VAS] pain score, flexion, range of motion [ROM], use of a mobility aid and stiffness) and at 1 year (2011 KSS, FJS, VAS, flexion and ROM). **Results**: There were no statistical significant differences in 2011 KSS or FJS at 1 year post-operatively between the two groups. Multivariate analysis showed no independent association of either technique with the 1-year follow-up KSS Objective Knee Indicators score (adjusted beta coefficient (aβ) = −2.371 [−7.380; 2.638], *p* = 0.357), KSS Patient Satisfaction score (aβ = −2.522 [−6.887; 1.842], *p* = 0.262), KSS Patient Expectations score (aβ = 0.629 [−0.928; 2.186], *p* = 0.431), KSS Functional Activities score (aβ = −3.399 [−10.881; 4.082], *p* = 0.377) or 1-year follow-up FJS (aβ = −5.168 [−19.887; 9.550], *p* = 0.494). **Conclusions:** There were no significant differences between the FA and rKA/load sensor groups in the 2011 KSS or FJS at 1 year post-operatively. To our knowledge, this is the first study to compare clinical outcomes between robotic-assisted FA TKA and rKA TKA. Clinical outcomes in the rKA/sensor group were similar to previous studies using rKA without robotic assistance or a load sensor. This was also the first report of the clinical outcomes of FA. The results need to be validated by larger scale studies to avoid potential type 2 errors.

## 1. Introduction

Total knee arthroplasty (TKA) is an effective procedure for symptomatic end-stage knee arthritis [1,2] with good clinical and survivorship outcomes [3,4]. However, up to 50% of patients report dissatisfaction following TKA [5,6,7,8]. Although the precise reasons for the poor patient satisfaction are not clear, successful TKA is dependent on several technical factors, including appropriate alignment of components and ligamentous balance of the knee joint [9,10,11,12]. Recent studies have suggested that suboptimal limb alignment may adversely affect post-operative knee biomechanics and kinematic function [13,14,15]. Moreover, one third of early TKA revisions are related to soft tissue imbalance, presenting as instability or stiffness [10,12,16]. Improved TKA pain outcomes have been reported when joint gaps are balanced throughout flexion [17]. However, there has been some debate regarding optimal alignment strategies and the most appropriate way to achieve ligament balance in TKA. Two personalised techniques to perform TKA have emerged, i.e., restricted kinematic alignment (rKA) and functional alignment (FA).

rKA is a hybrid option based on mechanical alignment (MA) and kinematic alignment (KA) first reported by Vendittoli in 2011, which involves reproducing the patient’s pre-arthritic anatomy and requires minimal intra-operative ligament release, thus avoiding reproduction of extreme anatomy [18]. This technique uses an algorithm to adjust more extreme patient anatomy that may be unsuitable for long-term implantation [13]. However, KA fails to achieve balanced TKA in almost 50% of cases [19,20], and the use of a restricted KA technique requires either soft tissue release and/or bone re-cutting to achieve balance in 30% of cases [21]. Therefore, Bardou-Jacquet et al. developed a robotic-assisted technique combining the rKA protocol with a load sensor for reliable ligament balancing using only bone re-cuts with a robotic arm [22].

FA is a recent technique first described by Haddad that developed after the advent of surgical aids, such as robotic-assisted TKA [23]. It constitutes a further development of the KA concept with increased precision. This technique has been shown to achieve balanced mediolateral soft tissue tension through the arc of knee flexion, as assessed using intra-operative load sensor technology, with minimal requirement for soft tissue release [24]. This study was performed to compare robotic-assisted FA with robotic-assisted rKA plus a load sensor.

The main objective of this prospective study was to compare the 2011 Knee Society Score (KSS) and Forgotten Joint Score (FJS) between FA robotic-assisted TKA and the combination of rKA/load sensor robotic-assisted TKA at 1 year after surgery. The secondary objectives were to compare visual analogue scale (VAS) pain scores, flexion, range of motion (ROM), the use of a mobility aid and stiffness between the two treatment groups at the 1-month follow-up, as well as the VAS score, flexion and ROM at the 1-year follow-up. Our hypothesis was that there would be no significant difference between these two techniques.

## 2. Methods

### 2.1. Patient Selection

This prospective, comparative and non-randomised study was performed at a robotic-assisted arthroplasty centre. The study population consisted of patients with end-stage knee arthritis undergoing robotic-assisted primary TKA between June 2020 and June 2021, with the FA or rKA/load sensor protocol. We excluded patients with post-traumatic, septic or inflammatory arthritis of the knee, body mass index (BMI) > 40 kg/m^2^, age > 90 years, advanced neoplastic, neurological or systemic disease that could alter functional scores, the requirement for reoperation on the prosthetic knee for any reason, and follow-up of <1 year. A total of 48 patients underwent FA robotic-assisted TKAs, performed by two authors who frequently apply this technique to their patients, while 51 underwent rKA/sensor robotic-assisted TKAs performed by one author who frequently applies this technique to their patients. Patients were therefore included in each group depending on which surgeon was performing the surgery. There was no randomization. Of these 99 patients, 4 were lost to follow-up (3 in the FA group and 1 in the rKA/sensor group). Furthermore, two patients in the FA group were excluded (one with post-traumatic peri-prosthetic patella fracture requiring reoperation due to painful pseudarthrosis and one with post-traumatic peri-prosthetic tibia fracture requiring casting), and three were excluded in the rKA/sensor group (two diagnosed with metastatic neoplasia requiring chemotherapy and one with neurological deficits of the lower limbs following a vertebra facture). Therefore, the finally study population consisted of 43 patients in the FA group and 47 in the rKA/sensor group.

The data were collected during pre- and post-operative consultations by the surgeon who performed TKA. The new KSS consists of four separate components: an Objective Knee Indicators score completed by the surgeon, and Patient Satisfaction, Patient Expectations and Functional Activities scores completed by the patient. A recent study analysed Patient Acceptable Symptom State (PASS) FJS data following primary TKA at 1 year post-operatively, and classified FJS < 33.3 as a poor outcome and FJS > 77.1 as a good outcome [25].

The patient demographics, pre-operative clinical parameters and patient-reported outcome measures (PROMs) are shown in Table 1 and Table 2. There were no significant differences in baseline or pre-operative characteristics between groups, with the exception of pre-operative flexion, which was 129.30 ± 8.44° (112.0°; 150.00°) in the FA group and 136.19 ± 8.93° (110.00°; 155.00°) in the rKA/sensor group (*p* < 0.001).

All patients provided informed consent during pre-operative consultations. The study protocol was approved by the Research Ethics Committee of the University Hospital of Research Ethics Committee of Bordeaux (Reference CER-BDX-2022-27).

### 2.2. Surgical Technique

#### 2.2.1. Both Treatment Groups

The cementless Triathlon knee system (Stryker Corp., Mahwah, NJ, USA) with a Tritanium baseplate, fixed condylar-stabilised (CS) bearing sparing the posterior cruciate ligament (PCL), uncemented femoral implant and cemented patellar resurfacing were used in both groups. All patients underwent surgery via a trans-quadricipital approach with medial para-patellar arthrotomy for TKA without a tourniquet, and routine soft tissue release was not performed during the approach or exposure.

All operative procedures were performed using a Mako robotic arm interactive orthopaedic system (Stryker Corp.). This system has been shown to be more accurate than manual techniques [26], thus causing less soft tissue damage [27], and can deliver the plan as intended with an accuracy of approximately 1° [28].

There were no cases of pin site infection, fracture or soft tissue damage, and no cases of robotic-assisted surgery were converted to manual TKA due to intra-operative complications.

#### 2.2.2. FA Group

FA uses a robotic platform that provides real-time 3D feedback to the surgeon on the implant position and limb alignment, as well as virtual flexion and extension gaps. Component positions were initially planned to achieve neutral mechanical limb alignment, and the implant position was then adjusted intra-operatively to restore the plane and obliquity of the joint as dictated by the soft tissue.

Planning software associated with the Mako robotic arm has allowed the development of a pre-resection balancing technique. This enables the assessment of soft tissue laxity and adjustment of the initial plan to achieve balanced soft tissue through the alteration of component alignment.

Intra-operative medial and lateral joint gaps in knee flexion and extension were initially assessed with the components in their planned positions, to achieve neutral mechanical limb alignment. Then, medial and lateral gaps were quantified in maximum knee extension and 90° flexion while applying valgus and varus forces to restore native tension in the medial and lateral soft tissue structures, respectively. Retractors must be removed from the surgical field at this point, and the patella is placed in the reduced position. The extension space is assessed using external manoeuvres to reduce deformity, and the flexion space is assessed using “spacer spoons” until the corrected position is achieved. To avoid over- or under-correction of the joint line, the spacer spoon should open the compartment without changing the contralateral tibiofemoral space. These two positions are thus ‘captured’ by the robotic system.

The effects of the planned bony cuts and implant positioning on the captured joint positions were displayed on the computer interface, allowing pre-emptive changes to be made based on the desired alignment and tibiofemoral gaps. Subsequently, the virtual 3D plan is modified by dynamic gap balancing to adjust the implant position. A thickness of 18 mm in flexion and extension was required in the lateral and medial compartments (global thickness of the implant). Lower targets were set for the extension gap in cases of hyperextension (recurvatum), and higher targets were set for cases of fixed-flexion deformity.

The femoral component was planned perpendicular to the mechanical axis of the femur and parallel to the transepicondylar axis (TEA), which was externally rotated by approximately 3° relative to the posterior condylar axis (PCA) [29,30]. In the sagittal plane, the femoral component was set to 0–5° of flexion to optimise implant positioning and prevent notching. The femoral component could then be rotated to within 3° of the TEA to balance the flexion gap, and manipulated to within 3° in the coronal plane to balance the extension gap. In the coronal plane, the tibial component was perpendicular to the tibial mechanical axis, to keep the extension and flexion gaps within 3° of varus. In the sagittal plane, the tibial component position matched the patient’s pre-arthritic posterior tibial slope, and was modified to balance the flexion gap if necessary. In the axial plane, the tibial component was positioned using the line of Akagi [29,30]. The largest tibial implant size that did not overhang the anteroposterior or mediolateral bone was selected. The arithmetic hip–knee–ankle (aHKA) was controlled to within 180 ± 3°. JLO was restored via valgus correction of the distal femoral resection and varus correction of the proximal tibial resection. By avoiding over-resection of the distal femur, the height of the joint line was maintained, avoiding the potential problem of mid-flexion instability associated with raising of the joint line [31,32]. Similarly, avoidance of under-resection of the distal femur avoided the need to compensate for a tight extension gap by using a thinner polyethylene insert, although this induced flexion instability. No ligament release was performed.

Once the knee had been virtually balanced using the mako robotic system (V2, Stryker, Kalamazoo, MI, United States), robotic arm-assisted surgery was performed to accurately replicate the plan, resulting in a balanced TKA.

#### 2.2.3. rKA/Load Sensor Group

Planning was based on pre-operative CT and the principles of rKA, as defined by Vendittoli [33,34]. The ‘safe range’ was defined as independent tibial and femoral cuts within ±5° of the bone’s neutral mechanical axis (i.e., JLO coronal alignment within ±5° of neutral) and an aHKA within 3° of neutral, with femoral anatomy preservation prioritised.

**Figure 1:**
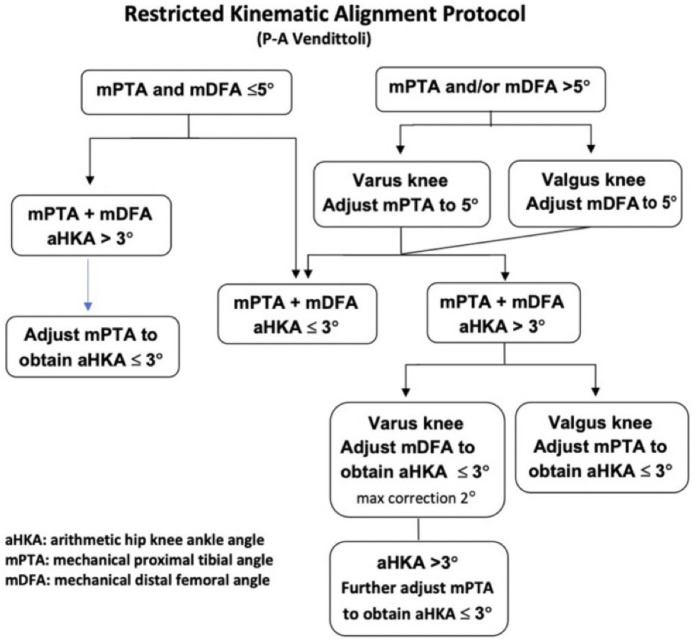
rKA protocol [35].

The rKA algorithm (Figure 1) advocates correcting the bone contributing most to the deviation of alignment. In most mild varus knees, the tibia is the main contributor, whereas it is the femur in valgus cases. In more extreme cases (e.g., aHKA > 10°), both the femur and tibia contribute to the anatomy (i.e., severe varus with the femur and tibia in varus). In such varus cases, we limit the femoral anatomy modification to 2°, while in severe valgus cases, after reducing the mean distal femoral angle to 5°, no further modification of the femur was performed. The tibia had to be within 2° of varus to keep the overall aHKA within ±3°.

After planning, collateral ligaments were tensioned ahead of the first bone cuts in millimetre increments using metal bone paddles, to compensate for cartilage wear. The position of the implant was then adjusted on the interface, to obtain a constant 18 mm space between the femur and tibia in extension and 90° flexion. Bone cutting was performed using the Mako robotic arm, and trial implants were then placed. To achieve balance, the VERASENSE load sensor (Stryker Corp.) was then positioned between the trial implants. The sensor has a microprocessor and an integrated nanosensor system allowing the transmission of data to a portable graphical display.

**Figure 2:**
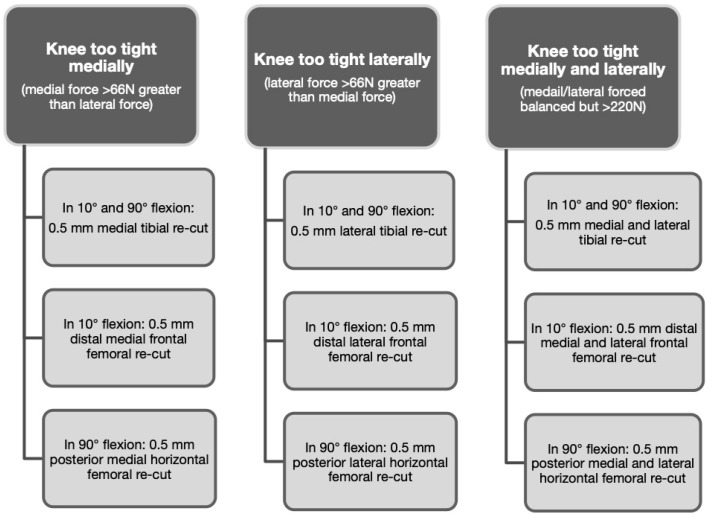
Bone re-cut algorithm [36].

**Figure 3:**
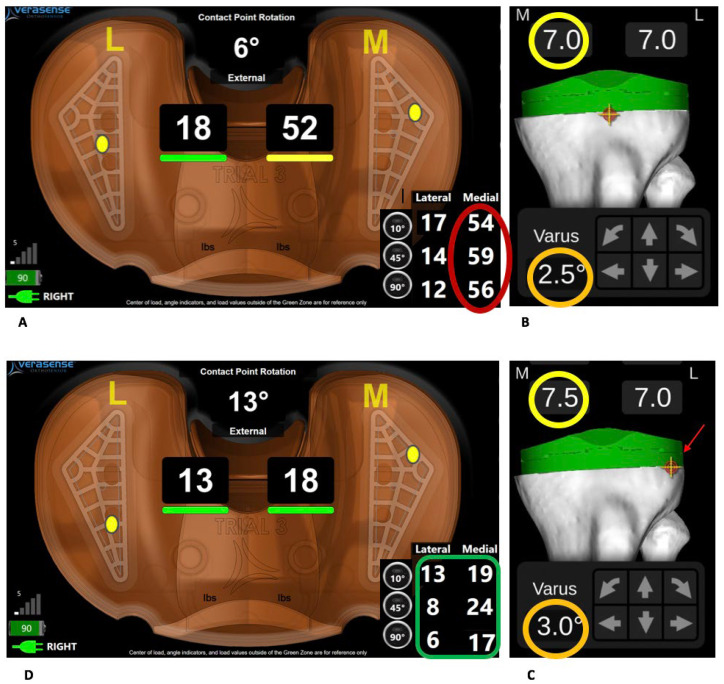
Tibial re-cut for a medially tight knee in flexion and extension. (**A**) The sensor shows >15 lbf difference between the medial and lateral compartments in 10° and 90° flexion. (**B**) Trial implant positioning according to the first bone cuts; yellow circle: cut bone thickness (mm), and orange circle: tibial varus in frontal plane. (**C**) To reduce medial force in flexion and extension, the tibia is further cut by 0.5 mm medially in the frontal plane (yellow circle); the centre of rotation of the cut plane is shifted laterally (red arrow) and 0.5° varus is added to the frontal cut plane (orange circle). (**D**) The knee is balanced, with medial-lateral difference < 15 lbf.

The sensor measured and localised the peak load on the medial and lateral tibiofemoral interfaces. The arthrotomy was closed and sensor data were acquired at 90° and 10° flexion without varus or valgus constraint, with one hand under the thigh and the other under the heel. The knee balance criteria were those of Gustke et al. [16]: a difference between the lateral and medial side < 66 Newtons (N) (i.e., <15 pound force [lbf]), with a single compartmental pressure of ≤45 lbf). Based on sensor-derived data displayed on the graphical user interface, only bone re-cuts were performed according to the algorithm shown in Figure 2. Re-cutting was performed using the robotic arm 3D interface, half-millimetre by half-millimetre, with load sensor checking between cuts (Figure 3). Once balance was achieved, the patella was resurfaced and the final implants were then introduced.

Only 47% (22/47) of knees were balanced in terms of both flexion and extension after the placement of trial implants, as measured by sensor-guided technology and with the application of Gustke’s criteria [16]. Sixteen patients underwent tibial re-cutting, five had a femoral distal re-cut and four had a femoral posterior re-cut. After performing ligament balancing by bone re-cutting, 89% (42/47) of knees were balanced. Of the five patients in whom balancing was unsuccessful, two had a femoral distal re-cut, one had a femoral posterior re-cut and two had a tibial re-cut. The surgeon tolerated these imbalances because the values obtained after bone re-cutting were close to the threshold value. There were no cases in which ligament release was performed.

### 2.3. Statistical Analysis

The recorded variables were summarised using descriptive statistics (median with range for continuous variables and frequency with proportion for categorical data). Qualitative variables were compared using the Chi-square test (or Fisher’s exact test as appropriate) and the Wilcoxon rank sum test (nonparametric test) was used for univariate analysis. Linear regression was performed to determine the individual effects of the technique (i.e., rKA/sensor vs. FA), baseline demographics (i.e., age and BMI) and pre-operative knee parameters (i.e., flexion, maximum flexion, and knee morphotype) on the short-term functional scores (i.e., KSS and FJS) at the 1-year follow-up, with independent risk estimates reported as the adjusted beta coefficient (aβ). Moreover, logistic regression analysis was performed to determine the individual effects of the technique (i.e., rKA/sensor vs. FA) on the FJS subgroups (i.e., FJS < 33.3, FJS 33.3–77.1 and FJS > 77.1), with independent risk estimates reported as the adjusted odds ratio (OR). Finally, missing variables were imputed using multiple imputation with the rms package, which employs a combination of additive regression, bootstrapping and predictive mean matching. In all analyses, *p* < 0.05 was taken to indicate statistical significance. Statistical analyses were performed using R software (version 3.3.2; R Foundation for Statistical Computing, Vienna, Austria). As this was a prospective exploratory cohort study comparing two emerging surgical techniques, no a priori sample size calculation was performed. To aid the interpretation of non-significant findings in the primary outcomes, a post hoc power analysis was conducted for the primary between-group comparisons.

## 3. Results

### 3.1. Outcomes at the 1-Month Follow-Up

There were no statistically significant differences between the FA and rKA/sensor groups in VAS score (2.2 ± 1.4 vs. 2.36 ± 1.69, *p* = 0.986), ROM (99.2 ± 13.6° vs. 96.2 ± 13.4°, respectively, *p* = 0.245), stiffness (7 [17%] vs. 11 [25%], *p* = 0.430) or use of a walking aid (n = 9 [21%] with one crutch and n = 0 [0%] with two crutches vs. n = 13 [28%] with one crutch and n = 2 [4%] with two crutches, *p* = 0.336).

Flessum was significantly different between the FA and rKA/sensor groups (3.50 ± 5.98° vs. 0.45 ± 2.11°, *p* < 0.001) (Table 3) without it being clinically meaningful, as 3° of difference is minimal.

### 3.2. Outcomes at the 1-Year Follow-Up

There were no statistically significant differences in the 2011 KSS or FJS at 1 year post-operatively (vs. baseline) in either group. Furthermore, there were no significant differences in VAS score, flexion or ROM in either group at 1 year (Table 4). Patient expectations showed a decline from pre- to post-operatively in both the FA group (from 13.8 ± 1.5 to 10.5 ± 3.1) and rKA/sensor group (from 14.2 ± 1.74 to 10.4 ± 3.2).

Multivariate analysis found no significant independent association of either technique with the 1-year follow-up KSS Objective Knee Indicators score (adjusted beta coefficient (aβ) = −2.371 [−7.380; 2.638], *p* = 0.357), KSS Patient Satisfaction score (aβ = −2.522 [−6.887; 1.842], *p* = 0.262), KSS Patient Expectations score (aβ = 0.629 [−0.928; 2.186], *p* = 0.431), KSS Functional Activities score (aβ = −3.399 [−10.881; 4.082], *p* = 0.377), 1-year follow-up total FJS (aβ = −5.168 [−19.887; 9.550], *p* = 0.494), FJS < 33.3 (OR = 0.668 [0.128; 3.475], *p* = 0.632), FJS 33.3–77.1 (OR: 1.974 [0.803; 4.851], *p* = 0.138), or FJS > 77.1 (OR = 0.537 [0.123; 2.339], *p* = 0.408) (Table 5 and Table 6).

However, in linear regression analysis, age was negatively associated with the KSS *Functional Activities* score at the 1-year follow-up (aβ = −0.441 [−0.829; −0.053], *p* = 0.029) (Table 5). High BMI was independently associated with a poor FJS result (<33.3; OR = 1.756 [0.577; 0.991], *p* = 0.020) (Table 6).

## 4. Discussion

The main objective of this study was to compare the 1-year follow-up PROMs (i.e., 2011 KSS and FJS) between two emerging techniques used to reproduce the constitutional alignment and ligament balance. There were no significant differences in 2011 KSS or FJS at 1 year after surgery between the FA and rKA/sensor groups.

KA fails to achieve balanced TKA in almost 50% of cases, which is often attributed to an increased flexion gap and varus deformity [19,20]. Moreover, the rKA technique requires soft tissue release and/or bone re-cutting to achieve balance in 30% of cases [21]. Therefore, Bardou-Jacquet et al. recently described an original technique using rKA to avoid reproducing extreme anatomy that could compromise long-term survivorship, combined with a load sensor positioned between the trial implants guiding bone re-cuts with a robotic arm to achieve reliable and reproducible ligament balancing [22]. Although clinical outcomes of rKA have been reported previously, this is the first report of the clinical results of rKA achieved using a robotic arm and load sensor. A previous study of 100 cementless rKA TKAs (Triathlon System; Stryker, Kalamazoo, Michigan, United States), performed with computer-assisted navigation (OrthoMap; Stryker) without a load sensor, reported a mean FJS of 65.9 ± 29.6 at a mean follow-up of 49 months (range: 32–60 months) [38]. In our study, the 1-year post-operative mean FJS in the rKA/sensor group was 64.3 ± 28.2. Thus, the technique using a combination of robotic-assisted rKA/load sensor produced similar results to rKA with computer-aided navigation without a load sensor, but the results are achieved at an earlier stage after surgery (i.e., at 12 and 49 months, respectively). MacDessi et al. reported a 1-year post-operative mean FJS of 63.9 ± 26.6 in 70 rKA TKAs performed using computer-assisted navigation (OrthoMap; Stryker) and a load sensor (VERASENSE; Stryker) to achieve ligament balance (by soft tissue release and/or bone re-cutting) [21]. Abhari et al. reported a mean JFS of 72 ± 27.0 at a mean follow-up of 17 months (range: 11–27 months) in 115 TKAs performed with an rKA protocol with robotic assistance (Mako; Stryker), but without a load sensor [39]. These observations suggest that using a robotic arm to perform ligament balancing by bone re-cutting, guided by the load sensor, did not improve the results of rKA compared to the technique using only robotic assistance or only a load sensor. In comparison, MacDessi et al. reported a 1-year post-operative mean FJS of 56.8 ± 26.0 in 68 MA TKAs with computer-assisted navigation (OrthoMap; Stryker) and a load sensor (VERASENSE; Stryker) [21].

In our study, flexion and/or extension imbalances were found in 53% of knees in the rKA/sensor group, consistent with previous studies [19,20], but this was reduced to 11% after applying the bone re-cutting protocol. Only one patient required an increase of +2 mm in polyethylene thickness, suggesting that this technique does not result in overcutting. The clinical benefit of a load sensor is debatable; it achieved a balanced knee in 85–95% of cases in previous studies [40,41,42]. Some studies suggested improvement of clinical outcomes in procedures using a sensor [16,40,43] while others showed no significant benefit [41,42]. However, in those studies, ligament balancing was performed using ligament release and/or bone re-cutting, and bone re-cuts were carried out without robotic assistance. To accurately assess the clinical impact of the load sensor, it would be interesting to compare both groups of TKAs with alignment performed with and without a load sensor, in terms of the ability to achieve reproducible ligament balancing by bone re-cutting with a robotic arm.

### 4.1. Alignment Strategies

FA in TKA was developed to restore the native joint line height, obliquity and knee kinematics, with computer-assisted technology applied to guide bone resection and implant positioning. The goal is to implant the components in a position that restores the plane and obliquity of the joint with minimal damage to the soft tissue. A previous study showed that mediolateral soft tissue balance, as measured by sensor-guided technology, can be consistently achieved by FA with robotic assistance, which adjusts bone resection and optimises implant positioning while minimising soft tissue release [24]. However, there have been no studies of the functional and clinical outcomes of this alignment technique, nor of implant survivorship, although a randomised controlled trial at University College London Hospital [23], and two more in Australia [44] and New Zealand [45] are currently underway to compare MA robotic-assisted TKA with FA robotic-assisted TKA. The three trials differ in terms of the surgical alignment limits, balancing algorithm and use of assistive technology. Alterative applications of FA have been described [46] that come closer to the rKA limits. The combined results of these trials may help to determine the ideal way to perform FA. In this study, components were pre-operatively planned in neutral MA and adjusted intra-operatively to achieve FA. Further studies are needed to determine whether the initial component position should be planned in KA and subsequently adjusted intra-operatively to achieve FA.

To our knowledge, this is the first study to compare the clinical outcomes of robotic-assisted FA TKA with robotic-assisted rKA TKA. This originality is one of the strengths of this study. The small sizes of the groups in our study could explain the lack of differences between these techniques, i.e., the statistical power required to detect clinically significant differences may have been lacking. Further studies with larger cohorts, particularly including patients with large deformities, are required to validate our results. These two techniques consider constitutional alignment but differ in their surgical alignment limits; they aim to achieve a balance between flexion and extension in two different ways that can produce the same results, especially in cases with small deformities. The 1-month follow-up flexion was lower in our rKA/sensor than the FA group (0.45 ± 2.11° vs. 3.50 ± 5.98°, *p* < 0.001), but this result was not clinically relevant.

Post-operatively, a decline in the KSS Patient Expectations score was observed in both groups and 13% of all patients (12/90) obtained poor results on the FJS. This rate was lower than that of patient dissatisfaction (20%) reported in most previous studies [1,5,6], but suggests that despite the availability of personalised techniques and use of advanced technologies, we are still unable to fully satisfy patient expectations. Therefore, other avenues must be explored to improve patient outcomes. There is currently a paucity of data and no scientific consensus on sagittal phenotypes of the knee joint, which should take possible physiological extension deficits or hyperextension into account [47,48]. Moreover, we must be aware of the complex phenotypes of the native trochlea groove of the knee joint with respect to its mediolateral positioning, which is relevant for positioning of the prosthetic femoral component. Most TKA systems were originally designed for MA, in which the femoral component is externally rotated. Using these components with personalised techniques, neutral or internal femoral rotation with a flush anterior femoral cut may under- or overstuff the native trochlea, depending on the discordance between the patient’s anatomy and implant design, leading to anterior knee pain with different aetiologies [49,50].

### 4.2. Limitations

This study had several limitations. Firstly, the groups were not comparable with respect to pre-operative maximum flexion, resulting in selection bias. The difference in pre-operative flexion can be explained by the fact that the data were acquired during the procedure using robotic navigation, with application of maximum passive flexion to the knee by the surgeon. However, the force applied to the knee could differ between surgeons. These differences between groups were taken into account in multivariate analysis. The relatively small sample size may have limited the ability to detect small-to-moderate between-group differences. A post hoc power analysis was therefore performed for the primary outcomes. With a total sample of 90 patients (43 in the FA group and 47 in the rKA/load sensor group) and assuming a two-sided α level of 0.05, the study had approximately 16% power to detect a small effect size (Cohen’s d = 0.20), 65% power to detect a moderate effect size (d = 0.50), and 96% power to detect a large effect size (d = 0.80). The minimal detectable standardised effect size with 80% power was approximately d = 0.60. These findings indicate that while the study was adequately powered to detect large differences between techniques, it was underpowered to exclude the presence of small-to-moderate true differences. Consequently, the absence of statistically significant differences in the primary outcomes should be interpreted in conjunction with effect estimates and confidence intervals, and larger studies are needed to confirm equivalence between techniques. Secondly, clinical outcomes and objective scores were collected by the surgeon who performed TKA without blinding, which may have introduced information bias. However, among the scales used in this study, only the KSS Objective Knee Indicators was completed by the surgeon; the other instruments (FJS and KSS Patient Expectations, Patient Satisfaction and Patient Function scores) were completed directly by the patient in the waiting room before consultation. Third, procedures were performed by different surgeons, which may have introduced bias in terms of the interpretation of results. The gender imbalance (*p* = 0.111) may also influence PROMs. Then, the study design is prospective but non-randomised, with surgeon-dependent allocation. This introduces major selection and performance bias. Finally, only the short-term results of the techniques were described in this preliminary report. Further mid- and long-term analyses of clinical outcomes and implant survivorship are necessary.

### 4.3. Conclusions

In conclusion, there were no significant differences between FA and the combined rKA/load sensor technique for robotic-assisted TKA, in terms of the 2011 KSS and FJS at 1 year post-operatively. The surgical technique combining restricted kinematic alignment (rKA) with a load sensor provides comparable results to the FA technique and can be used routinely.

## Figures and Tables

**Table 1:** Baseline demographic characteristics.

Variables	Overall N = 90	Groups		
		FA n = 43	rKA & Sensor n = 47	*p* Value
**Gender, n (%)**				0.111
Male	54 (60%)	30 (70%)	24 (51%)	
Female	36 (40%)	13 (30%)	23 (49%)	
**Age at time of surgery, m ± SD (range)**	70.06 ± 9.31	70.21 ± 8.01	69.91 ± 10.53	0.882
	(47.00; 91.00)	(48.00; 85.00)	(47.00; 91.00)	
**Side, n (%)**				0.520
Right	46 (51%)	24 (56%)	22 (47%)	
Left	44 (49%)	19 (44%)	25 (53%)	
**BMI (kg/m^2^), m ± SD (range)**	28.13 ± 3.74	27.88 ± 2.96	28.36 ± 4.39	0.542
	(18.70; 39.50)	(22.80; 35.90)	(18.70; 39.50)	
**Lifestyle, n (%)**				1.000
Living with a partner	81 (90%)	39 (91%)	42 (89%)	
Living alone	9 (10%)	4 (9%)	5 (11%)	
**Walking aid, n (%)**				0.515
None	75 (83%)	38 (88%)	37 (79%)	
One crutch	12 (13%)	4 (9%)	8 (17%)	
Two crutches	3 (4%)	1 (3%)	2 (4%)	
**OD (min), m ± SD (range)**	74.12 ± 12.19	74.74 ± 14.3	73.57 ± 10.25	0.967
	(50.00; 120.00)	(50.0; 120.00)	(56.00; 90.00)	
**Rehabilitation, n (%)**				1.000
Ambulatory	78 (87%)	37 (86%)	41 (87%)	
Rehabilitation centre	12 (13%)	6 (14%)	6 (13%)	

**SD**, standard deviation; **m**, mean; and **n**, number. **BMI**, body mass index, and **OD**, operative duration.

**Table 2:** Comparaison of preoperative anatomical and clinical parameters.

Variables	Overall N = 90	Groups		
		FA n = 43	rKA & Sensor n = 47	*p* Value
**Pre-op VAS score, m ± SD (range)**	6.99 ± 1.29	7.14 ± 1.26	6.85 ± 1.32	0.259
0–10 none to worst	(5.00; 10.00)	(5.00; 10.00)	(5.00; 10.00)	
**Pre-op flessum (°), m ± SD (range)**	5.37 ± 4.98	5.38 ± 4.66	5.36 ± 5.32	0.785
	(0.00; 20.00)	(0.0; 16.00)	(0.0; 20.00)	
**Pre-op flexion max (°), m ± SD (range)**	133.15 ± 9.27	129.30 ± 8.44	136.19 ± 8.93	**<0.001**
	(110.00; 155.00)	(112.0; 150.00)	(110.00; 155.00)	
**HKA (°), m ± SD (range)**	175.49 ± 5.94	175.59 ± 6.28	175.4 ± 5.76	0.884
	(160.00–189.00)	(163.00; 189.00)	(160.00; 189.00)	
**Knee morphotype, n (%)**				0.299
Neutral (180 ± 3°)	27 (30.00%)	14 (32%)	13 (28%)	
Varus				
3–10°	42 (47%)	16 (37%)	26 (55%)	
>10°	14 (15%)	8 (19%)	6 (13%)	
Valgus				
3–10°	7 (8%)	5 (12%)	2 (4%)	
>10°	0	0	0	
**Controlateral knee state, n (%)**				0.466
No OA	34 (38%)	14 (33%)	20 (43%)	
OA	36 (40.00%)	20 (46%)	16 (34%)	
Arthroplasty	20 (22%)	9 (21%)	11 (23%)	
**Pre-op clinical outcome score m ± SD (range)**				
KSS				
Objective score	47.21 ± 15.55	46.64 ± 16.78	47.72 ± 14.7	0.892
0–100 worst to best	(10.00; 83.00)	(10.00; 83.00)	(28.00; 82.00)	
Expectations score	13.97 ± 1.65	13.76 ± 1.54	14.15 ± 1.74	0.450
0–15 worst to best	(6.00; 15.00)	(9.00; 15.00)	(6.0; 15.00)	
Satisfaction score	13.30 ± 6.54	2.81 ± 5.81	13.74 ± 7.23	0.691
0–40 worst to best	(4.00; 32.00)	(0; 17.00)	(4.0; 32.00)	
Functional score	41.24 ± 14.99	38.98 ± 12.98	43.26 ± 16.6	0.183
;0–100 worst to best	(1.00; 79.00)	(6.0; 68.00)	(1.0; 79.00)	

**SD**, standard deviation; **m**, mean; and **n**, number. **VAS**, visual analogue scale; **HKA**, hip-knnee-ankle angle; **KSS**, Knee Society Knee Scoring System; and **OA**, osteoarthritis.

**Table 3:** Outcomes at the 1-month follow-up.

Variables	Overall N = 90	Groups		
		FA n = 43	rKA & Sensor n = 47	*p* Value
**VAS score, m ± SD (range)**	2.31 ± 1.56	2.24 ± 1.45	2.36 ± 1.69	0.986
0–10 none to worst	(0.00; 7.00)	(0.00; 5.00)	(0.00; 7.00)	
**Flessum (°), m ± SD (range)**	1.94 ± 4.64	3.50 ± 5.98	0.45 ± 2.11	<0.001 *
	(0.00; 30.00)	(0.00; 30.00)	(0.00; 10.00)	
**ROM (°), m ± SD (range)**	97.66 ± 13.38	99.17 ± 13.56	96.23 ± 13.36	0.245
	(60.00; 124.00)	(60.00; 124.00)	(60.0; 120.00)	
Stiffness **, n (%)	18 (21%)	7 (17%)	11 (25%)	0.430
**Walking aid, n (%)**				0.336
None	66 (73%)	34 (79%)	32 (68%)	
One crutch	22 (25%)	9 (21%)	13 (28%)	
Two crutches	2 (2%)	0	2 (4%)	

**SD**, standard deviation; **m**, mean; **n**, number; **VAS**, visual analogue scale; and **ROM**, range of motion. * Statistically significant. ** Defined by a physical examination with extension limited to 15° short of full extension or flexion < 90° [37].

**Table 4:** Outcomes at the 1-year follow-up.

Variables	Overall N = 90	Groupes		
		FA n = 43	rKA & Sensor n = 47	*p* Value
**VAS score, m ± SD (range)**	0.72 ± 1.56	0.618 ± 1.26	0.839 ± 1.86	0.973
0–10 none to worst	(0.00; 7.00)	(0.00; 5.00)	(0.00; 7.00)	
**Flessum (°), m ± SD (range)**	0.57 ± 1.83	0.735 ± 2.18	0.37 ± 1.33	0.564
	(0.00; 10.00)	(0.00; 10.00)	(0.00; 5.00)	
**ROM (°), m ± SD (range)**	117.87 ± 8.57	119.76 ± 7.37	115.38 ± 9.65	0.078
	(95.00; 135.00)	(100.0; 135.00)	(95.0; 131.00)	
**Post-op scores**				
**KSS, m ± SD (range)**				
**Objective score**	90.83 ± 10.04	91.97 ± 10.53	89.42 ± 9.64	0.078
0–100 worst to best	(62.00; 100.00)	(62.00; 100.00)	(63.00; 100.00)	
**Expectations score**	10.43 ± 3.10	10.47 ± 3.09	10.4 ± 3.17	0.851
0–15 worst to best	(3.00; 15.00)	(3.00; 15.00)	(4.00; 15.00)	
**Satisfaction score**	31.80 ± 8.45	31.28 ± 8.98	32.51 ± 7.89	0.680
0–40 worst to best	(6.00; 40.00)	(6.00; 40.00)	(6.00; 40.00)	
**Functional score**	77.79 ± 14.93	79.23 ± 12.82	76.72 ± 16.53	0.768
0–100 worst to best	(28.00; 99.00)	(53.00; 99.00)	(28.00; 98.00)	
**FJS**				
**Total, m ± SD (range)**	65.25 ± 27.57	66.51 ± 27.5	64.26 ± 28.18	0.627
0–100 worst to best	(4.20; 100.00)	(11.50; 100.00)	(4.20; 100.0)	
**Subgroups, n (%)**				0.926
FJS < 33.3	12 (13%)	6 (14%)	6 (13%)	
Bad result				
FJS between 33.3 and 77.1	29 (32%)	13 (30%)	16 (34%)	
Intermediate result				
FJS > 77.1	49 (55%)	24 (56%)	25 (53%)	
Good result				

**SD**, standard deviation; **m**, mean; **n**, number; **VAS**, visual analogue scale; **ROM**, range of motion; **KSS**, Knee Society Knee Scoring System; and **FJS**, Forgotten Joint Score.

**Table 5:** Factors associated with the 1-year follow-up KSS.

	KSS_O			KSS_S			KSS_E			KSS_F		
	aβ Coef	IC95	*p*	aβ Coef	IC95	*p*	aβ Coef	IC95	*p*	aβ Coef	IC95	*p*
**TECHNIQUE**												
** FA**		**Reference**			**Reference**			**Reference**			**Reference**	
** rKA & sensor**	−2.371	[−7.380; 2.638]	0.357	−2.522	[−6.887; 1.842]	0.262	0.629	[−0.928; 2.186]	0.431	−3.399	[−10.881; 4.082]	0.377
**DEMOGRAPHIC**												
**Age**	0.189	[−0.070; 0.449]	0.159	−0.210	[−0.436; 0.016]	0.074	−0.066	[−0.147; 0.014]	0.112	**−0.441**	**[−0.829; −0.053]**	0.029 *
**BMI**	0.099	[−0.525; 0.725]	0.756	0.568	[0.023; 1.113]	0.055	0.160	[−0.033; 0.355]	0.101	0.729	[−0.204; 1.663]	0.131
**KNEE** **PARAMETERS**												
**Pre-op flessum**	−0.298	[−0.774; 0.177]	0.223	0.415	[0.001; 0.830]	0.054	0.123	[−0.024; 0.271]	0.106	0.008	[−0.702; 0.719]	0.981
**Pre-op maximum flexion**	0.085	[−0.227; 0.399]	0.593	0.127	[−0.145; 0.400]	0.364	−0.066	[−0.164; 0.030]	0.183	−0.113	[0.581; 0.354]	0.636
**Knee morphotype**												
Neutral		**Reference**			**Reference**			**Reference**			**Reference**	
Varus 3–10°	1.074	[−8.059; 10.209]	0.818	−7.029	[−14.989; 0.931]	0.088	−1.758	[−4.598; 1.081]	0.229	−6.155	[−19.800; 7.489]	0.380
Varus > 10°	−12.676	[−27.959; 2.606]	0.109	−13.077	[−26.396; 0.240]	0.059	−3.338	[−8.089; 1.413]	0.173	−8.144	[−30.973; 4.684]	0.487
Valgus 3–10°	0.883	[−9.589; 11.355]	0.869	2.329	[−6.796; 11.455]	0.619	2.678	[−0.577; 5.934]	0.112	1.832	[−13.810; 17.470]	0.819

**aβ Coef**, adjusted Beta coefficient; **IC95**, confidence interval; ***p***, *p*-value; **FA**, functional alignment; **rKA**, restricted kinematic aligment; and **BMI**, body mass index. * Statistically significant.

**Table 6:** Factors associated with the 1-year follow-up FJS.

	FJS Total			FJS < 33.3			33.3 ≤ FJS ≤ 77.1			FJS > 77.1		
	aβ Coef	IC95	*p*	OR	IC95	*p*	OR	IC95	*p*	OR	IC95	*p*
**TECHNIQUE**												
** FA**		**Reference**			**Reference**			**Reference**			**Reference**	
** rKA & sensor**	−5.168	[−19.887; 9.550]	0.494	0.668	[0.128; 3.475]	0.632	1.974	[0.803; 4.851]	0 138	0.537	[0.123; 2.339]	0.408
**DEMOGRAPHIC**												
**Age**	−0.465	[−1.228; 0.299]	0.237	1.013	[0.931; 1.102]	0.763	0.908	[0.784; 1.053]	0.205	1.003	[0.933; 1.078]	0.921
**BMI**	1.664	[−0.173; 3.500]	0.081	**1.756**	**[1.577; 1.991]**	0.020 *	1.666	[1.082; 2.564]	0.200	0.916	[0.761; 1.104]	0.360
**KNEE** **PARAMETERS**												
**Pre-op Flessum**	0.528	[−0.870; 1.930]	0.462	0.866 [0.733; 1.022]		0.089	1.700 [1.193; 2.422]		0.330	0.927 [0.811; 1.060]		0.271
**Pre-op maximum flexion**	0.419	[−0.501; 1.340]	0.375	1.024 [0.922; 1.138]		0.647	0.905 [0.761; 1.077]		0.263	1.011 [0.930; 1.100]		0.788
**Knee morphotype**												
Neutral		**Reference**			**Reference**			**Reference**			**Reference**	
Varus 3–10°	−20.073	[−46.914; 6.770]	0.148	1.226	[0.062; 24.399]	0.893	0.800	[0.281; 2.270]	0.675	0.267	[0.019; 3.654]	0.323
Varus > 10°	−27.262	[−72.168; 17.600]	0.239	0.083	[<0.001; 35.699]	0.421	1.111	[0.286; 4.306]	0.878	0.087	[0.002; 6.258]	0.263
Valgus 3–10°	5.542	[−25.228; 36.300]	0.725	0.837	[0.013; 52.737]	0.932	2.666	[0.488; 14.558]	0.257	1.117	[0.060; 20.663]	0.940

**aβ Coef**, adjusted Beta coefficient; **OR**: Odd ratio; **IC95**, confidence interval; ***p***, *p*-value; **FA**, functional alignment; **rKA**, restricted kinematic aligment; and **BMI**, body mass index. * Statistically significant.

## Data Availability

The data presented in this study are available on request from the corresponding author.

## Abbreviations

TKA	total knee arthroplasty
FA	functional alignment
KS	Knee Society Score
FJS	Forgotten Joint Score
rKA	restricted kinematic alignment
MA	mechanical alignment
VAS	visual analogue scale
ROM	range of motion
CS	condylar-stabilised
PCL	posterior cruciate ligament
aHKA	arithmetic hip-knee-ankle
TEA	trans-epicondylar axis

## References

[1] Bourne R.B., Chesworth B.M., Davis A.M., Mahomed N.N., Charron K.D.J. (2010). Patient satisfaction after total knee arthroplasty: Who is satisfied and who is not?. Clin. Orthop. Relat. Res..

[2] Bourne R.B., McCalden R.W., MacDonald S.J., Mokete L., Guerin J. (2007). Influence of patient factors on TKA outcomes at 5 to 11 years followup. Clin. Orthop. Relat. Res..

[3] Font-Rodriguez D.E., Scuderi G.R., Insall J.N. (1997). Survivorship of cemented total knee arthroplasty. Clin. Orthop. Relat. Res..

[4] Rodricks D.J., Patil S., Pulido P., Colwell C.W. (2007). Press-fit condylar design total knee arthroplasty. Fourteen to seventeen-year follow-up. J. Bone Jt. Surg. Am..

[5] Nam D., Nunley R.M., Barrack R.L. (2014). Patient dissatisfaction following total knee replacement: A growing concern?. Bone Jt. J..

[6] Baker P.N., van der Meulen J.H., Lewsey J., Gregg P.J., National Joint Registry for England and Wales (2007). The role of pain and function in determining patient satisfaction after total knee replacement: Data from the National Joint Registry for England and Wales. J. Bone Jt. Surg. Br..

[7] Milner C.E. (2009). Is gait normal after total knee arthroplasty? Systematic review of the literature. J. Orthop. Sci..

[8] Bourne R.B., Chesworth B., Davis A., Mahomed N., Charron K. (2010). Comparing patient outcomes after THA and TKA: Is there a difference?. Clin. Orthop. Relat. Res..

[9] Bozic K.J., Kurtz S.M., Lau E., Ong K., Chiu V., Vail T.P., Rubash H.E., Berry D.J. (2010). The epidemiology of revision total knee arthroplasty in the United States. Clin. Orthop. Relat. Res..

[10] Fehring T.K., Odum S., Griffin W.L., Mason J.B., Nadaud M. (2001). Early failures in total knee arthroplasty. Clin. Orthop. Relat. Res..

[11] Lonner J.H., Siliski J.M., Scott R.D. (1999). Prodromes of failure in total knee arthroplasty. J. Arthroplast..

[12] Sharkey P.F., Hozack W.J., Rothman R.H., Shastri S., Jacoby S.M. (2002). Insall Award paper. Why are total knee arthroplasties failing today?. Clin. Orthop. Relat. Res..

[13] Almaawi A.M., Hutt J.R.B., Masse V., Lavigne M., Vendittoli P.-A. (2017). The Impact of Mechanical and Restricted Kinematic Alignment on Knee Anatomy in Total Knee Arthroplasty. J. Arthroplast..

[14] Bellemans J., Colyn W., Vandenneucker H., Victor J. (2012). The Chitranjan Ranawat award: Is neutral mechanical alignment normal for all patients? The concept of constitutional varus. Clin. Orthop. Relat. Res..

[15] Cherian J.J., Kapadia B.H., Banerjee S., Jauregui J.J., Issa K., Mont M.A. (2014). Mechanical, Anatomical, and Kinematic Axis in TKA: Concepts and Practical Applications. Curr. Rev. Musculoskelet. Med..

[16] Gustke K.A., Golladay G.J., Roche M.W., Elson L.C., Anderson C.R. (2014). A new method for defining balance: Promising short-term clinical outcomes of sensor-guided TKA. J. Arthroplast..

[17] Wakelin E.A., Shalhoub S., Lawrence J.M., Keggi J.M., De Claire J.H., Randall A.L., Ponder C.E., Koenig J.A., Lyman S., Plaskos C. (2021). Improved total knee arthroplasty pain outcome when joint gap targets are achieved throughout flexion. Knee Surg. Sports Traumatol. Arthrosc..

[18] Hutt J.R.B., LeBlanc M.-A., Massé V., Lavigne M., Vendittoli P.-A. (2016). Kinematic TKA using navigation: Surgical technique and initial results. Orthop. Traumatol. Surg. Res..

[19] Shatrov J., Batailler C., Sappey-Marinier E., Gunst S., Servien E., Lustig S. (2022). Kinematic alignment fails to achieve balancing in 50% of varus knees and resects more bone compared to functional alignment. Knee Surg. Sports Traumatol. Arthrosc..

[20] Lee G.-C., Wakelin E., Plaskos C. (2022). What Is the Alignment and Balance of a Total Knee Arthroplasty Performed Using a Calipered Kinematic Alignment Technique?. J. Arthroplast..

[21] MacDessi S.J., Griffiths-Jones W., Chen D.B., Griffiths-Jones S., Wood J.A., Diwan A.D., Harris I.A. (2020). Restoring the constitutional alignment with a restrictive kinematic protocol improves quantitative soft-tissue balance in total knee arthroplasty: A randomized controlled trial. Bone Jt. J..

[22] Bardou-Jacquet J., Murgier J., Laudet F., Fabre T. (2021). Combining load sensor and robotic technologies for ligament balance in total knee arthroplasty. Orthop. Traumatol. Surg. Res..

[23] Kayani B., Konan S., Tahmassebi J., Oussedik S., Moriarty P.D., Haddad F.S. (2020). A prospective double-blinded randomised control trial comparing robotic arm-assisted functionally aligned total knee arthroplasty versus robotic arm-assisted mechanically aligned total knee arthroplasty. Trials.

[24] Chang J.S., Kayani B., Wallace C., Haddad F.S. (2021). Functional alignment achieves soft-tissue balance in total knee arthroplasty as measured with quantitative sensor-guided technology. Bone Jt. J..

[25] Singh V., Fiedler B., Huang S., Oh C., Karia R.J., Schwarzkopf R. (2022). Patient Acceptable Symptom State for the Forgotten Joint Score in Primary Total Knee Arthroplasty. J. Arthroplast..

[26] Hampp E.L., Chughtai M., Scholl L.Y., Sodhi N., Bhowmik-Stoker M., Jacofsky D.J., Mont M.A. (2019). Robotic-Arm Assisted Total Knee Arthroplasty Demonstrated Greater Accuracy and Precision to Plan Compared with Manual Techniques. J. Knee Surg..

[27] Khlopas A., Chughtai M., Hampp E.L., Scholl L.Y., Prieto M., Chang T.-C., Abbasi A., Bhowmik-Stoker M., Otto J., Jacofsky D.J. (2017). Robotic-Arm Assisted Total Knee Arthroplasty Demonstrated Soft Tissue Protection. Surg. Technol. Int..

[28] Sires J.D., Wilson C.J. (2021). CT Validation of Intraoperative Implant Position and Knee Alignment as Determined by the MAKO Total Knee Arthroplasty System. J. Knee Surg..

[29] Aglietti P., Sensi L., Cuomo P., Ciardullo A. (2008). Rotational position of femoral and tibial components in TKA using the femoral transepicondylar axis. Clin. Orthop. Relat. Res..

[30] Akagi M., Oh M., Nonaka T., Tsujimoto H., Asano T., Hamanishi C. (2004). An anteroposterior axis of the tibia for total knee arthroplasty. Clin. Orthop. Relat. Res..

[31] Luyckx T., Vandenneucker H., Ing L.S., Vereecke E., Ing A.V., Victor J. (2018). Raising the Joint Line in TKA is Associated with Mid-flexion Laxity: A Study in Cadaver Knees. Clin. Orthop. Relat. Res..

[32] Stambough J.B., Edwards P.K., Mannen E.M., Barnes C.L., Mears S.C. (2019). Flexion Instability After Total Knee Arthroplasty. J. Am. Acad Orthop. Surg..

[33] Vendittoli P.-A., Blakeney W. (2017). Redefining knee replacement. Orthop. Traumatol. Surg. Res..

[34] Blakeney W., Beaulieu Y., Kiss M.-O., Rivière C., Vendittoli P.-A. (2019). Less gap imbalance with restricted kinematic alignment than with mechanically aligned total knee arthroplasty: Simulations on 3-D bone models created from CT-scans. Acta Orthop..

[35] Vendittoli P.-A., Martinov S., Blakeney W.G. (2021). Restricted Kinematic Alignment, the Fundamentals, and Clinical Applications. Front. Surg..

[36] Bardou-Jacquet J., Murgier J., Laudet F. Combining sensors and robotic technologies to balance TKA. Proceedings of the European Society of Sports Traumatology, Knee Surgery & Arthroscopy (ESSKA) 20th Annual Meeting.

[37] Healy W.L., Della Valle C.J., Iorio R., Berend K.R., Cushner F.D., Dalury D.F., Lonner J.H. (2013). Complications of Total Knee Arthroplasty: Standardized List and Definitions of The Knee Society. Clin. Orthop. Relat. Res..

[38] Laforest G., Kostretzis L., Kiss M.-O., Vendittoli P.-A. (2022). Restricted kinematic alignment leads to uncompromised osseointegration of cementless total knee arthroplasty. Knee Surg. Sports Traumatol. Arthrosc..

[39] Abhari S., Hsing T.M., Malkani M.M., Smith A.F., Smith L.S., Mont M.A., Malkani A.L. (2021). Patient satisfaction following total knee arthroplasty using restricted kinematic alignment. Bone Jt. J..

[40] Golladay G.J., Bradbury T.L., Gordon A.C., Fernandez-Madrid I.J., Krebs V.E., Patel P.D., Suarez J.C., Rueda C.A.H., Barsoum W.K. (2019). Are Patients More Satisfied with a Balanced Total Knee Arthroplasty?. J. Arthroplast..

[41] Wood T.J., Winemaker M.J., Williams D.S., Petruccelli D.T., Tushinski D.M., de Beer Jde V. (2021). Randomized Controlled Trial of Sensor-Guided Knee Balancing Compared to Standard Balancing Technique in Total Knee Arthroplasty. J. Arthroplast..

[42] MacDessi S.J., Wood J.A., Diwan A., Harris I.A., on behalf of the SENSOR BALANCE Study Group (2022). Intraoperative pressure sensors improve soft-tissue balance but not clinical outcomes in total knee arthroplasty: A multicentre randomized controlled trial. Bone Jt. J..

[43] Chow J.C., Breslauer L. (2017). The Use of Intraoperative Sensors Significantly Increases the Patient-Reported Rate of Improvement in Primary Total Knee Arthroplasty. Orthopedics.

[44] Steer R., Tippett B., Khan R.N., Collopy D., Clark G. (2021). A prospective randomised control trial comparing functional with mechanical axis alignment in total knee arthroplasty: Study protocol for an investigator initiated trial. Trials.

[45] Young S.W., Zeng N., Tay M.L., Fulker D., Esposito C., Carter M., Bayan A., Farrington B., Van Rooyen R., Walker M. (2022). A prospective randomised controlled trial of mechanical axis with soft tissue release balancing vs functional alignment with bony resection balancing in total knee replacement—A study using Stryker Mako robotic arm-assisted technology. Trials.

[46] Shatrov J., Battelier C., Sappey-Marinier E., Gunst S., Servien E., Lustig S. (2022). Functional Alignment Philosophy in Total Knee Arthroplasty—Rationale and technique for the varus morphotype using a CT based robotic platform and individualized planning. SICOT J..

[47] Freeman Ma R., Pinskerova V. (2005). The movement of the normal tibio-femoral joint. J. Biomech..

[48] Pinskerova V., Samuelson K.M., Stammers J., Maruthainar K., Sosna A., Freeman Ma R. (2009). The knee in full flexion: An anatomical study. J. Bone Jt. Surg. Br..

[49] Rivière C., Dhaif F., Shah H., Ali A., Auvinet E., Aframian A., Cobb J., Howell S., Harris S. (2018). Kinematic alignment of current TKA implants does not restore the native trochlear anatomy. Orthop. Traumatol. Surg. Res..

[50] Rivière C., Iranpour F., Harris S., Auvinet E., Aframian A., Parratte S., Cobb J. (2018). Differences in trochlear parameters between native and prosthetic kinematically or mechanically aligned knees. Orthop. Traumatol. Surg. Res..

